# iPSCs as a Platform for Disease Modeling, Drug Screening, and Personalized Therapy in Muscular Dystrophies

**DOI:** 10.3390/cells8010020

**Published:** 2019-01-03

**Authors:** Jose L. Ortiz-Vitali, Radbod Darabi

**Affiliations:** Center for Stem Cell and Regenerative Medicine (CSCRM), The Brown Foundation Institute of Molecular Medicine for the Prevention of Human Diseases (IMM), The University of Texas Health Science Center at Houston, Houston, TX 77030, USA; Jose.L.OrtizVitali@uth.tmc.edu

**Keywords:** iPSCs, muscular dystrophies, DMD, duchenne, gene correction, CRISPR/Cas9, stem cells

## Abstract

Induced pluripotent stem cells (iPSCs) are the foundation of modern stem cell-based regenerative medicine, especially in the case of degenerative disorders, such as muscular dystrophies (MDs). Since their introduction in 2006, many studies have used iPSCs for disease modeling and identification of involved mechanisms, drug screening, as well as gene correction studies. In the case of muscular dystrophies, these studies commenced in 2008 and continue to address important issues, such as defining the main pathologic mechanisms in different types of MDs, drug screening to improve skeletal/cardiac muscle cell survival and to slow down disease progression, and evaluation of the efficiency of different gene correction approaches, such as exon skipping, Transcription activator-like effector nucleases (TALENs), Zinc finger nucleases (ZFNs) and RNA-guided endonuclease Cas9 (CRISPR/Cas9). In the current short review, we have summarized chronological progress of these studies and their key findings along with a perspective on the future road to successful iPSC-based cell therapy for MDs and the potential hurdles in this field.

## 1. Introduction

Since 2006, and with the generation of induced pluripotent stem cells (iPSCs) [1,2,3], the field of stem cell biology and regenerative medicine has developed a more optimistic outlook to providing personalized patient therapy using stem cells. Due to their patient-specific nature and ease of generation, iPSCs are a great candidate for disease modeling, drug screening, and gene correction. iPSCs also carry other major advantages, such as reduced ethical concern (unlike embryonic stem cells (ESCs)), the ability to give rise to every cell type (pluripotency), and better immunocompatibility since they are harvested and reprogrammed from the same patient [4,5,6]. Initially, iPSCs were derived via integrating viral vectors that delivered the key transcription factors *Klf4*, *Oct3/4*, *Sox2*, *Lin-28*, and c-Myc [1,2,3,7], but this posed a concern for downstream therapeutic use due to unforeseen insertional mutagenesis caused by viral genome integration. To overcome this problem, new methods were developed, such as non-integrating viral (Sendai virus) and non-viral methods (cDNA, mRNA, protein) that are much safer than the traditional method, and alleviate the risk of insertional mutagenesis [8,9,10,11,12,13]. These advances, in conjunction with the possibility of the generation of tissue-specific cells from iPSCs and new gene correction techniques, such as TALENs [14,15] and CRISPR/Cas9 [16,17], create great hopes for personalized stem cell therapy to become a reality for treating countless degenerative disorders.

One such group of disorders that would benefit from iPSC-based personalized therapy are the muscular dystrophies (MDs). MDs are a heterogeneous group of degenerative skeletal muscle disorders caused by a variety of genetic defects that lead to progressive muscle wasting, formation of fibrotic and adipose tissue in the muscle, and even death in severe cases due to cardiomyopathy and respiratory failure [18,19,20]. There are many types of MDs with various degrees of severity, such as Becker (BMD), myotonic, limb girdle (LGMD), and the most well-known, Duchenne Muscular Dystrophy (DMD). While mild to moderate MDs become symptomatic at young or middle age with manageable complications, severe types, unfortunately, can be fatal [18,19,20,21]. An example of a severe type of muscular dystrophy is Duchenne Muscular Dystrophy (DMD), which occurs primarily in males between three and five years old and leads to progressive muscle wasting and atrophy [18,21,22]. By the teenage years, these patients are limited to wheelchairs and suffer from severe respiratory and cardiac problems. Unfortunately, to date, there is no definitive cure for MDs and treatment is limited to the management of complications by surgery and medical care for cardiac and respiratory complications [18,19,20]. Because iPSC and gene editing bringing new hope for the treatment of muscular dystrophies, we have overviewed the chronological development of promising advances (see Supplementary Figure S1) using the technologies for MD disease modeling and gene correction. We point out major caveats and give future directions.

## 2. iPSCs and Disease Modeling for Muscular Dystrophies

Many animal models, such as mouse, pig, and dog, are currently available to study the biology of MDs, in particular for DMD [23,24,25]. However, they can fall short of the recapitulation of disease pathology and phenotype due to the variability of biochemical abnormalities and compensatory mechanisms in different species. While dog and pig models prove to be useful DMD animal models by providing clinically relevant data, gestation periods are considerably longer. Further, the animals require much larger spaces to house. Both of these are factors lead to the limitation of the power of significance and greater expense [26]. On the other hand, while mice models do not have these problems, it is still difficult to extrapolate mice data to DMD patients due to the presence of compensatory mechanisms and milder phenotypes (such as *mdx* mice).

In contrast, iPSCs provide an optimal tool for fast expansion and creation of tissue-specific cells to model diseases in vitro. These patient-specific cells can be used for a variety of purposes including drug screening, in vitro analysis of true myogenic capacity, and disease pathology before the onset of the typical secondary effects of inflammation. As seen in modeling, a disease-affected cell type with iPSCs also facilitates testing parameters, such as myoblasts derived from DMD patients or animal models, drug toxicity, dose-response, and efficacy on the target. In addition, mice or other animal models may not capture the true genetic variation of the human population (in-breeding) or simply have different phenotypes in muscle stem cells’ behavior and regeneration due to their different genetic background [27]. iPSCs derived in a patient-specific manner, however, bypass this issue and maintain as close a model as possible to the true genetic nature of the human population. Therefore, the skeletal muscle field began to use iPSCs for disease modeling soon after the development of iPSC reprogramming in 2007 (listed in Table 1).

Initial reports in 2008 and later were mainly focused on the generation of iPSCs from MDs (such as DMD and LGMD) and proof of concept for their myogenic differentiation [28,29,30,31]. After this step, subsequent studies used iPSCs for disease modeling and evaluation of pathophysiology in muscular dystrophies such as Facioscapulohumeral muscular dystrophy (FSHD), myotonic dystrophy, and DMD [32,33,34]. These are typical examples of using iPSCs to model the disease and identify pathologic features or etiologies. The first study successfully proved the pathologic role of DUX4 in inhibition of mesoderm induction and its potential role as a neural-inducing factor, and as a potential contributing factor to FSHD pathology [32]. The second study used iPSCs from myotonic dystrophy type 1 (DM1) and demonstrated involved mechanisms in CTG·CAG triplet repeat expansion in the 3′ untranslated region of the DMPK1 gene in patient iPSCs [33]. In the third study, DMD iPSC-derived myotubes were used to study the abnormalities in calcium ion influx following electric stimulation and their reversal after dystrophin restoration, which is a classic example of iPSC disease modeling in the case of muscular dystrophies [34].

The iPSCs have also been used to model and study the mechanisms of dilated cardiomyopathy in DMD patients [35]. DMD iPSC-derived cardiomyocytes demonstrated major phenotypic and pathologic features of dilated cardiomyopathy, such as elevated levels of resting calcium, mitochondrial damage, and cell apoptosis. A similar study also used iPSCs from LGMD patients to study ion channel dysfunction in cardiomyocytes derived from patient iPSCs and successfully demonstrated the presence of its pathologic features, such as abnormal action potentials, differences in peak and late ion currents, and significant reduction of systolic and diastolic intracellular calcium concentrations [36].

Another interesting study has recently used DMD-iPSCs from patients with different dystrophin mutations to study variations in disease phenotype [37]. Using a directed differentiation approach for myogenic induction in iPSCs, derived myoblasts demonstrated typical disease-related phenotypes with patient-to-patient variability. These included variations in inflammatory response genes, increased BMP/TGF-β signaling, and reduced fusion potential of myotubes from different patients. Another example of using iPSCs for modeling muscular dystrophy is the recent report of the generation of a 3D platform using hydrogels to provide the right niche for proper muscle stem cell and myofiber development, as well as modeling skeletal muscle laminopathies and their pathologic features, such as nuclear abnormalities [38]. Finally, a recent study has used the DMD-iPSC-derived myotubes to study myotube alignment and orientation in the cell microenvironment and demonstrated the importance of Dystrophin-Associated-Protein-Complex (DAPC) in proper cell alignment through its interaction with laminin [39]. Overall, these efforts have so far been able to clearly demonstrate the great potential of iPSCs for disease modeling in the case of various types of MDs, as well as helping to identify new pathologic mechanisms involved in these disorders.

## 3. iPSCs and Drug Screen for Muscular Dystrophies

Since drug screening and development has been mostly dependent on animal models and immortalized cell lines, iPSC disease models now provide a unique human/patient platform. iPSC based-disease models can be generated in a patient-specific manner and will potentially be useful in the identification of involved pathologies as well as in developing new treatments for MDs. In addition, patient-specific iPSC drug screening not only allows for the study of disease pathways but also when paired with in vivo models and can allow for testing of the safety and efficacy of the new drug candidate.

Although the application of iPSCs for drug screening started in early 2009 with the modeling of cardiac arrhythmias [40], in the case of muscular dystrophies it was delayed until a few years later (listed in Table 2). One major reason for this delay might be due to difficulties in myogenic differentiation and expansion, which with the availability of recent methods, will be overcome in the near future [41,42].

The first report of using iPSCs for drug screening in MDs was published in 2014 when a team of investigators generated 16 iPSC lines from patients with Duchenne and Becker muscular dystrophies (DMD, BMD) [43]. This was done by utilizing in vitro myogenic induction using MyoD over-expression, and the effects of two hypertrophic proteins, IGF-1 and Wnt7a, were evaluated on patient iPSC-derived myogenic cells. The results indicated the effectiveness of both agents in inducing significant muscle hypertrophy in iPSC-derived myotubes from the patients. The results supported their potential application as a pharmacologic treatment in muscular dystrophies [43]. Later, in 2015, DMD-iPSC-derived cardiomyocytes (CMs) were used for modeling dilated cardiomyopathy and the efficacy of a membrane sealant, Poloxamer 188, was evaluated on the cells [35]. The results indicated a significant reduction in the resting cytosolic calcium level along with reduced caspase-3 (CASP3) activation and apoptosis in DMD iPSC-CMs, indicating the potential therapeutic benefit of this agent [35]. In another study, the effect of a nitric oxide donor and ATP-Sensitive Potassium Channel Opener, Nicorandil, was evaluated in DMD iPSC-CMs [44]. As DMD cardiomyocytes generally suffer from decreased levels of endothelial and neuronal nitric oxide synthases, they are more susceptible to cell injury and cell death after stress due to increased levels of reactive oxygen species (ROS) and dissipation of the mitochondrial membrane potential [44]. This iPSC-based screen confirmed the protective effect of Nicorandil through nitric oxide-cyclic guanosine monophosphate pathway and mitochondrial adenosine triphosphate-sensitive potassium channels. Interestingly, when used in isolated hearts of *mdx* mice (a mouse model for DMD), Nicorandil was able to improve the overall cardiac function [44], thus, indicating its potential cardioprotective effect as a therapeutic agent for the treatment of cardiomyopathy in MDs.

To facilitate drug screening for MDs, another recent study has adapted the myogenic differentiation of human iPSCs in a 384-well format with uniform and high myotube differentiation efficiency, which makes it very favorable for high-throughput drug screening (HTS) [45]. Finally, another interesting study has recently used the myotonic dystrophy DM1-iPSCs to model the disease and use antisense oligonucleotide treatment screen. The results indicated the effectiveness of the antisense oligonucleotide by abolishing RNA foci and rescuing BIN1 mis-splicing in DM1 iPS cell-derived myotubes [46]. Overall, these limited reports indicate the feasibility and usefulness of iPSCs for drug screening in the case of MDs and provide basic guidelines and protocols toward the development of HTS for myopathies using patient iPS cells. Of course, as mentioned above, any candidate for HTS in iPSC screening should be further validated for in vivo safety and efficacy using appropriate animal models.

## 4. iPSCs and Gene Correction for Muscular Dystrophies

With a lack of effective therapy for muscular dystrophies, gene-based therapies combined with iPSC technology provide an attractive approach to treatment. Therefore, multiple efforts have been made so far to validate the efficacy of gene correction strategies on iPSCs derived from MD patients (listed in Table 3). One of the initial reports using this strategy was done in 2010 through the application of a human artificial chromosome (HAC) carrying the complete genomic dystrophin sequence (DYS-HAC) [47]. After the generation of DMD-iPSCs, the vector was transferred to the cells via microcell-mediated chromosome transfer (MMCT). As proof of principle, tissue-specific expression of dystrophin was evaluated and confirmed in muscle tissues, as well as its proper expression in chimeric mice [47]. This was the pioneering report of gene correction in iPSCs in the case of muscular dystrophies.

Another popular approach for gene correction in the case of DMD involves application of exon-skipping to reconstitute a truncated form of the dystrophin gene. This employs an antisense oligonucleotide (AON) that binds to the mutation site in mRNA and induces exon-skipping, thus, allowing partial restoration of the reading frame. One example of this approach is the work undertaken in 2013 using exon-skipping to restore dystrophin expression in human DMD iPSC-derived cardiomyocytes [48]. Using seven different DMD iPSCs harboring various mutations across the DMD gene, an antisense oligonucleotide-mediated skipping of exon 51 was able to restore the dystrophin expression to nearly 30% of the normal level, thus, proving the relative efficiency of this strategy [48]. At the same time, another ex vivo gene therapy approach was developed with the delivery of the micro-utrophin (µUTRN) using the highly efficient “sleeping beauty” transposon system for gene transfer and stable gene expression [49]. In this study, dKO-iPSCs (from dKO mice, a double-knockout mouse model for dystrophin and utrophin with a severe dystrophic phenotype similar to DMD patients) were corrected, and the expression of the transgene was evaluated in vitro as well as in vivo after transplantation into the dystrophic mice. Engrafted cells were able to express high levels of micro-utrophin, along with muscle stem cell engraftment and functional improvement in muscle contractility [49].

The next advancement to this field was the use of site-specific gene editing technologies for correction of the mutations. A classic example of this approach is through the application of Transcription activator-like effector nucleases (TALENs) and RNA-guided endonuclease Cas9 (CRISPR/Cas9) for gene correction in DMD iPSCs, which was reported in 2015 [50]. By comparing three different corrections strategies (exon-skipping, frame-shifting, and exon knockin) in DMD-iPSCs, the efficacy of each approach was evaluated. Results indicated similar efficacy of TALEN and CRISPR/Cas9 for targeting, and superiority of exon knockin for the restoration of dystrophin when compared to exon-skipping and frame-shifting [50]. This work also proved the efficacy of programmable nucleases for gene correction in iPSCs from muscular dystrophy/DMD patients. Another interesting study in 2016 designed a single CRISPR/Cas9 deletion strategy that can target a majority of DMD patient mutations (60%) [51]. This strategy includes deletion and Non-homologous end joining (NHEJ) of the DMD gene around its common mutation hotspot to reframe the gene. Corrected DMD iPSCs demonstrated restored dystrophin expression after differentiation into cardiomyocytes and skeletal muscle as well as in vivo mice transplantation [51].

Next, seamless allele-specific correction strategies were developed using a single-stranded oligonucleotide CRISPR/Cas9-mediated approach [52]. Using LGMD2B/2D-iPSCs, the investigators used a short single-stranded oligonucleotide for homology-directed repair along with a CRISPR/Cas9 gene-editing system to enhance its targeting efficiency. This approach led to the precise and seamless correction of the specific alleles. Although the efficiency was lower compared to other targeting approaches, it was still able to correct the expression of missing proteins (dysferlin and alpha-sarcoglycan) in tissue and a site-specific manner [52].

Recently a newer version of the gene editing system, CRISPR/Cpf1, has been evaluated for gene correction in DMD iPSCs as well as in mice models [53]. By using this system along with exon-skipping or correction of the non-sense mutations in DMD iPSCs, dystrophin expression was restored, and differentiated cardiomyocytes demonstrated enhanced contractility. In addition, germline-corrected *mdx* mice demonstrated significant improvements in muscle histology, such as restoration of dystrophin expression along with a significant reduction of fibrosis and inflammation [53]. The results demonstrated high genome-wide targeting efficiency and specificity of the CRISPR/Cpf1 for gene editing in muscular dystrophies, particularly in the case of DMD.

Finally, in another interesting report by the same group, guide RNAs were screened to identify optimal gRNAs capable of targeting conserved RNA splice sites in 12 exons in the hotspot region of DMD gene [54]. This strategy allows for the skipping of the most common mutant or out-of-frame DMD exons within or nearby mutational hotspots. As proof of concept, this approach was evaluated in iPSCs from multiple DMD patients with large deletions, point mutations, or duplications. Results proved the efficiency of this unified approach for restoring dystrophin protein expression (in iPSC-derived cardiomyocytes) within the most common types of dystrophin gene abnormalities clustered in these hotspots [54].

## 5. Perspective for Therapeutic Application of iPSCs in Muscular Dystrophies

Currently, there is no definitive cure for muscular dystrophies, and therapies mostly consist of the management of the cardiopulmonary morbidities. Treatments, such as steroids, and physical/surgical therapies for increasing range of motion and stretches are simply palliative [18]. Although iPSCs provide a promising forecast for clinical trials in the future, this technology is nowhere near to being used in practice due to two major problems: (a) variability in the generation of pure and safe populations of cells, and (b) the identification of the ideal cell type and delivery route to target different body muscles in the case of muscular dystrophies. 

One major obstacle is the development of a safe and pure myogenic cell population from iPSCs. This traditionally has been done using gene-overexpression or directed differentiation using small molecules or cytokines [30,31,41,55,56,57,58]. Although both methods demonstrated promising advances in the generation of the myogenic cells from iPSCs, there are still a few major concerns. Regarding gene over-expression, the risk of insertional mutagenesis using viral vectors remains a concern. Genomic integration is needed for stable expression of the desired gene. However, it carries the risk of insertional mutagenesis and unforeseen alterations in the genome leading to side effects, such as increased risk of malignancies [59]. These risks can also be very variable due to virus copy number, cell type, and the over-expressed gene effect [60] Moreover, Lentiviruses might cause immune reactions leading to undesirable consequences, such as triggering inflammatory reactions [61]. On the other hand, for directed differentiation approaches, parallel production of unwanted and poorly differentiated cells might carry unforeseen consequences, such as the chance of tumogenesis following cell transplantation [62]. Development of more efficient myogenic induction or directed differentiation protocols along with the identification of specific markers to purify myogenic cells will eventually eliminate this obstacle, as we and others have recently reported [41,42,63].

In addition, although the use of programmable endonuclease systems, such as TALEN and CRISPR, were designed to identify and generate DNA strand breaks at the desired and specific location of the genome, there is always the risk of off-target side effects, such as indels/mutations associated with these systems [64,65]. This risk can potentially be reduced by modification of the endonucleases to enhance their binding specificity, such as using a double nickase system or modification of gRNA length in the case of CRISPR [66,67]. In addition, new and improved high-throughput sequencing and bioinformatics allow for faster whole genome screening on corrected iPSCs and will eventually help to identify safe cells for future clinical applications. Moreover, it is still not clear if these methods are able to efficiently target and correct the gene defect in muscle stem cells (satellite cells), which is critical for maintaining the longevity of the correction in the treated muscles. Therefore, more studies are warranted to solve this obstacle.

For strategies using antisense oligonucleotide (AON) for exon-skipping, the barriers to overcome for DMD therapy include instability of chemical structures, the efficacy of drug delivery, dose-related toxicity, and the risk of off-target effects [68]. Furthermore, in vivo AON efficacy has not proven as strong as in vitro, possibly due to diffusion problems with large molecules, particularly in damaged or fibrotic tissues. However, phase 3 trial of this method for DMD has shown limited efficacy but only in patients who maintain walking ability, not in severe cases [69].

Another major problem facing the application of iPSCs for the treatment of muscular dystrophies is the route of delivery. Since most types are muscular dystrophies involving various groups of skeletal muscle in the body, local delivery through intramuscular injection is not a practical option. Therefore, systemic routes of cell delivery would be the ultimate approach. These include intra-arterial or intravenous cell delivery. Since intravenous cell delivery suffers from cell entrapment during the first passage through organs (such as the lungs) [70] and may lead to unwanted side-effects and a significant reduction in cell quantity before reaching their target muscles, the intra-arterial route has been considered in the case of systemic muscle disorders and dystrophies. In this case, desirable cells should also be able to bind to the muscle endothelium and cross the barrier toward interstitial muscle space and eventually engraft into the damaged muscle fibers. 

Although promising research has been done to study the efficiency of the intra-arterial cell route in animal models of muscular dystrophies using mesoangioblasts [71,72], in the case of iPSC-derived myogenic cells, there are limited reports so far. One good example is the derivation of mesoangioblasts-like cells from human LGMD2D-iPSCs and their subsequent gene correction using lentiviral vector expressing human α-sarcoglycan [31]. Corrected cells were then transduced to myogenic cells using MyoD expression and then transplanted into a null mouse model for α-sarcoglycan using intra-arterial injection through femoral arteries. As proof of concept, cell engraftment was verified by time-course imaging and histology. The results indicated the ability of human iPSC-derived cells to engraft into the dystrophic muscle following intra-arterial delivery [31]. Similar results were also reported by our collaborators and ourselves regarding the intravenous and intra-arterial delivery of murine and human iPSC-derived myogenic cells into dKO and *mdx* mice models [49,73]. Overall, these reports provide proof of concept for the ability of systemic cell delivery in mice models of muscular dystrophies. However, the efficiency of engraftment in these reports is rather low, and further investigations are warranted to identify the ideal iPSC-derived myogenic cells capable of efficient muscle engraftment following systemic cell delivery. Another important aspect of these studies should also include the investigation of potential side-effects related to systemic cell distribution, such as entrapment and seeding outside the target tissue (muscle) which has the potential for unforeseen consequences.

iPSC, along with novel gene editing technology, has brought new insights and hopes for cell-based therapies in the case of degenerative genetic disorders, such as muscular dystrophies, and it would not be implausible to witness clinical trials in the years to come using corrected iPSCs as personalized therapies for muscular dystrophies.

## Figures and Tables

**Table 1:** List of key studies using Induced pluripotent stem cells (iPSCs) for disease modeling in case of muscular dystrophies.

Disease Type	Cell Type	Methodology	Purpose	Year	Reference
DMD/BMD	iPSC-human	Retroviral transduction of iPSC inducing factors	Myogenic potential of DMD/BMD iPSCs.	2008	Park et al. [28]
DMD/*mdx* mice	iPSC-mice	Myogenic differentiation of murine iPSCs using gene over-expression	Testing in vivo engraftment potential	2011	Darabi et al. [29]
DMD/NSG-*mdx* ^4Cv^	ES/iPSC-human	Gene Over-expression. TA injection of hES/iPSC in NSG-*mdx* ^4Cv^ mice	Functional restoration of dystrophin in *mdx* mice.	2012	Darabi et al. [30]
LGMD2D	iPSC-human	Retroviral transduction of fibroblast to iPSC and inducible MyoD expression. IM injection of cells	In vivo transplantation of corrected iPSC gave rise to striated a-sarcoglycan^+^ fibers	2012	Tedesco et al. [31]
FSHD	iPSC-human	Retroviral transduction of iPSC factors and EB differentiation	Role of DUX4 in myogenic inhibition and neural induction	2010	Snider et al. [32]
DM1	iPSC-human	iPSC generation and evaluation of CTG-CAG repeat length	Mechanism of CTG-CAG repeat in 3’UTR of DMPK1 gene	2013	Du et al. [33]
DMD	iPSC-human	Transfection of Doxycycline inducible MyoD plasmid. Electrical stimulation and fluorescent Ca^2+^ marker to visualize influx	Reversal in abnormality of Ca^2+^ ion influx following dystrophin restoration	2015	Shoji et al. [34]
DMD	iPSC-human	Patient-derived DMD iPSC generation. Electrophysical recording and Ca^2+^ transients images with CMOS camera	Pathologic features of cardiomyopathy	2015	Lin et al. [35]
LGMD	iPSC-human	iPSC generated and patch clamp performed for ion currents and Ca^2+^ transients measured via fluorescence	Abnormalities and pathologic features in ion channel function in patient iPSC-derived cardiomyocytes	2018	El-Battrawy et al. [36]
DMD	iPSC-human	DMD iPSC corrected with DYSTROPHIN-HAC transfection	Variations in disease related phenotypes between DMD patients	2016	Choi et al. [37]
DMD/LGMD BMD	iPSC-human	3D matrix differentiation to observe myofibers formation. Triple lineage constructs created with 70% myogenic cells and 30% vascular	Development of 3D hydrogel platform for muscle stem cell and myofibers formation	2018	Maffioletti et al. [38]
DMD	iPSC-human	Cells cultured on culture substrated with nanogrooves coated with Matrigel or Laminin to observe myotube alignment with and without DAPC-Laminin interaction.	Myotube alignment and orientation in microenvironment and importance of DAPC	2018	Xu et al. [39]

**Table 2:** List of key studies using iPSCs for drug screening in case of muscular dystrophies.

Type of Model	Purpose	Method of Development	Year	Reference
iPSC DMD/BMD	Induction of muscle hypertrophy in iPSC-derived myotubes. Validation of pharmacologic treatment in MDs	In vitro myogenic induction: overexpressed MyoD and evaluated the effect on IGF-1 and Wnt7a in patient iPSCs	2014	Abujarour et al. [43]
iPSC-DMD	Modeling dilated cardiomyopathy and efficacy of membrane sealant Poloxamer 188 Confirm protective effect of Nicorandil in *mdx* mice as cardioprotective agent	Directed differentiation on feeder MEF cells. Electrophysiological recording performed as well as Ca^2+^ transients imaged with CMOS camera	2015	Lin et al. [35]
iPSC-DMD	HTS in 384-well format for drug screening	Directed differentiation and MyoD induction via various methods including re-plating technique, feeder MEF cells, and feeder-free adapted	2017	Uchimura et al. [45]
iPSC-DM1	To model DM1 and evaluate effectiveness of oligonucleotide treatment	Inducible Pax7 cell line for differentiation to skeletal muscle	2018	Mondragon-Gonzalez et al. [46]

**Table 3:** List of key studies using iPSCs for gene correction in case of muscular dystrophies.

Gene Based Therapy	Delivery System	Cell Type	Application	Year	Reference
Human Artificial Chromosome (HAC) carrying dystrophin sequence	Microcelle-mediated chromosome transfer	DMD-iPSC	Gene correction in DMD iPSC	2010	Kazuki et al. [47]
Antisense oligonucleotide (AON)	Polyethylenimine (PEI) transfection	DMD-iPSC cardiomyocytes (7 types)	Exon skipping in DMD to restore dystrophin expression	2013	Dick et al. [48]
Micro-utrophin (µUTRN) delivery	“sleeping beauty” transposon system	dKO iPSCs from mouse model (severely dystrophic)	Partial expression of µUTRN and muscle stem cell engraftment for functional muscle improvement	2013	Filareto et al. [49]
TALEN and CRISPR/Cas9	Electroporation	DMD-iPSC	Comparing correction strategies: exon-skipping, frame-shifting, and exon knock-in	2015	Li et al. [50]
CRISPR/Cas9	Nucleofection	DMD-iPSC	Simple deletion strategy to target 60% of DMD mutations in patients.	2016	Young et al. [49]
Single strand oligo CRISPR/Cas9	Nucleofection	LGMD2B/2D-iPSC	Tissue and site-specific expression of dysferlin and α-sarcoglycan proteins	2016	Turan et al. [52]
CRISPR-Cpf1	Nucleofection	DMD-iPSC cardiomyocytes	Evaluate gene correction along with exon-skipping strategy for dystrophin restoration in cardiomyocytes.	2017	Zhang et al. [53]
CRISPR/Cas9	Nucleofection	DMD-iPSC and 3D iPSC engineered heart muscle	Exon-skipping of mutant and out-of-frame DMD exons at hotspot regions	2018	Long et al. [54]

## Supplementary Materials

The following are available online, Figure S1: Chronological development of induced pluripotent stem cells (iPSC)-based studies in the case of muscular dystrophies. Schematic image demonstrates timeline emergence of key studies using iPSCs for modeling the disease, drug screening, and gene correction in case of muscular dystrophies.
