# Supplementary material for: iPSCs as a Platform for Disease Modeling, Drug Screening, and Personalized Therapy in Muscular Dystrophies
Source: Cells. 2019 Jan 3;8(1):20. doi: 10.3390/cells8010020 (PMC6356384; doi:10.3390/cells8010020)
Supplement: Supplementary file 1 [file cells-08-00020-s001.pdf]

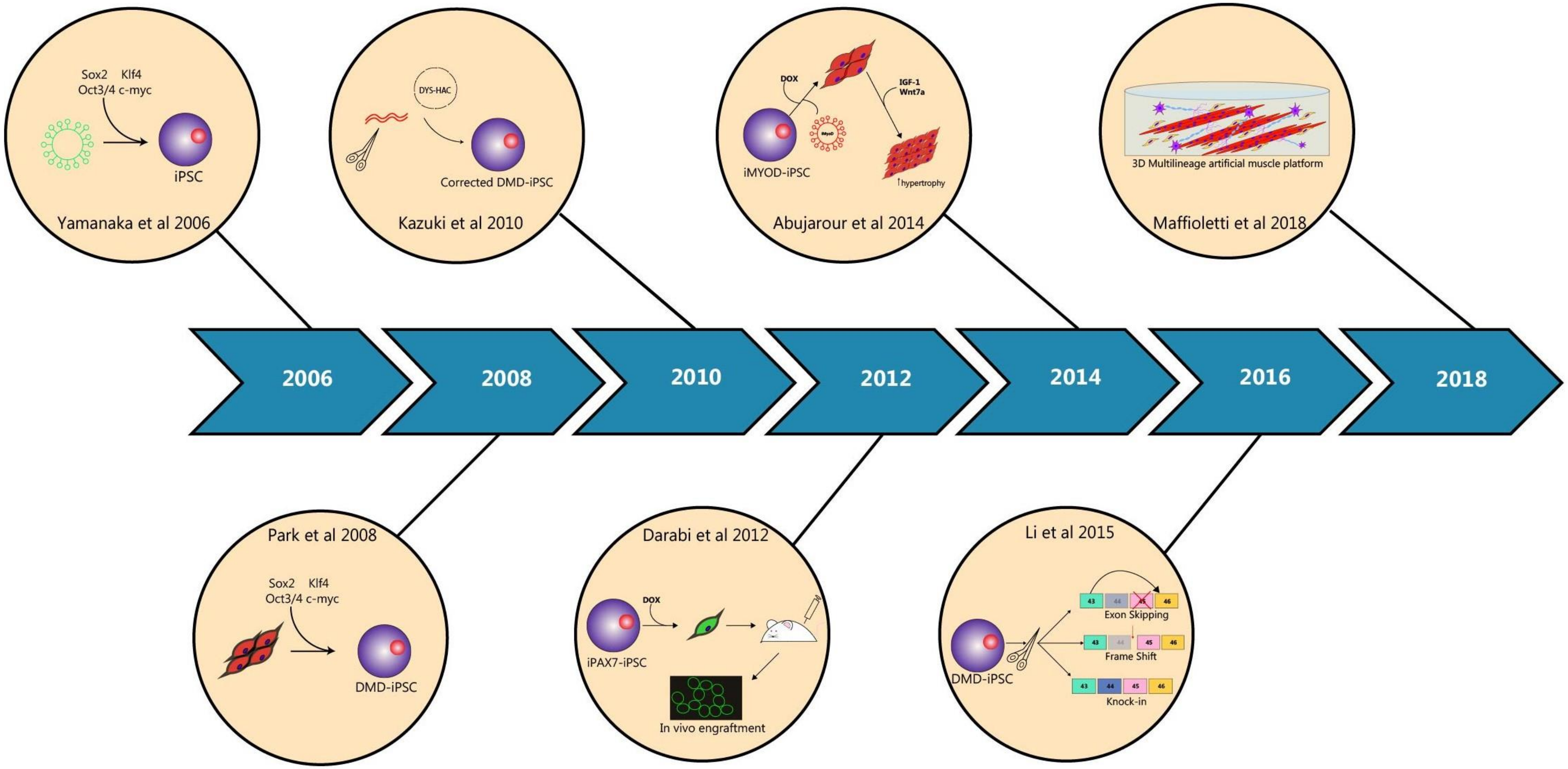

Figure S1. Chronological development of induced pluripotent stem cells (iPSC)-based studies in the case of muscular dystrophies. Schematic image demonstrates timeline emergence of key studies using iPSCs for modeling the disease, drug screening, and gene correction in case of muscular dystrophies.
